# Detection of Visually Imperceptible Blood Contamination in the Surgical Area Using Luminol Among Different Oral Surgical Procedures: An Observational Study

**DOI:** 10.7759/cureus.53821

**Published:** 2024-02-08

**Authors:** Kohila V Kannan, Saravanan Kandhasamy, Reena R John, Suresh Chinnakutti

**Affiliations:** 1 Oral and Maxillofacial Surgery, Vinayaka Mission’s Sankarachariyar Dental College, Vinayaka Mission’s Research Foundation, Salem, IND; 2 Oral and Maxillofacial Surgery, Vinayaka Mission's Sankarachariyar Dental College, Vinayaka Mission's Research Foundation, Salem, IND

**Keywords:** surgical site infections (ssi), blood splatter, luminol, oral surgery, blood contamination, infectious aerosols

## Abstract

Introduction

Oral surgeons often encounter a significant occupational risk of exposure to potentially harmful infectious diseases during minor oral surgical procedures. These diseases can be transmitted through direct contact with body fluids and aerosolized splatters that may not be visibly detectable. The likelihood of transmission is heightened for clinicians, healthcare workers, and patients alike. The reported prevalence of exposure to blood-borne infections in this field is as high as 90%, with half of these exposures being visually imperceptible.

Aim

The aim was to detect visually imperceptible blood contamination on personal protective equipment (PPE) and clinical surfaces using the chemiluminescence agent luminol during oral surgical procedures.

Materials and methods

Thirty minor oral surgical procedures were performed in the Oral and Maxillofacial Surgery Department after obtaining approval from the Institutional Ethics Committee (IEC), Vinayaka Mission’s Sankarachariyar Dental College, Vinayaka Mission’s Research Foundation, Salem, India. The surgeon, assistant, patient, and clinical surfaces (comprising 15 subsites within the surgical field) wore PPE. The PPE was scrutinized for traces of visually imperceptible blood contamination using luminol. The results of blood splatter on PPE and clinical surfaces in different oral surgical procedures between the non-aerosol and aerosol groups of different durations were analyzed statistically using the chi-square test with *p *< 0.05 considered significant.

Results

We observed that visually imperceptible blood contamination in non-aerosol procedures was detected on the assistant PPE kit (46.7%, *n* = 14), assistant face shield (40%, *n* = 12), suction apparatus (50%, *n* = 15), wall (30%, *n* = 9), and floor (56.7%, *n* = 17), in both aerosol and non-aerosol procedures. The *p-value* has been considered statistically significant at *p *< 0.05 between both the groups (aerosol and non-aerosol).

Conclusion

Our study results confirmed the presence of undetected blood spillage during aerosol procedures of 30 minutes and non-aerosol surgical procedures of more than 30 minutes over an area of 3.1 feet horizontally and 4.8 feet vertically. So, we strongly emphasize that PPE kits and face shields are mandatory for both surgeon and assistant while performing oral surgical procedures in order to prevent the risk of cross infections, proper infection prevention control protocol for the clinical surfaces also needs to be followed as a standard protocol in all operations.

## Introduction

Dental professionals (especially oral surgeons) frequently face blood contamination in the form of visually imperceptible aerosolized splatters during minor surgical procedures [1-5], exposing them to potentially infectious diseases such as hepatitis B (HBV) and C (HCV), human immunodeficiency virus (HIV), and airborne infections like COVID-19 [6], influenza, and pneumonia. These diseases can be transmitted through direct contact with body fluids and may persist on the surface for weeks. HBV and HCV can persist on surfaces and devices contaminated with blood for at least one and six weeks [7]. The prevalence of exposure [8-11] in the field is high with 90% due to blood-borne infections and 50% being invisible [12]. This study aims to detect visually imperceptible blood-contaminated areas and objects using the chemiluminescence agent luminol; ensuring proper sterilization and infection prevention control. To highlight in detail, this study aims to examine the visually imperceptible blood splatter on personal protective equipment (PPE) of surgeons and assistants, surgical field, and clinical surfaces.

## Materials and methods

This single-center observational study was conducted in the Department of Oral and Maxillofacial Surgery from March 2021 to March 2022 after obtaining Institutional Ethics Committee (IEC) approval from the Institutional Research Committee. The Ethical Committee of Vinayaka Mission’s Sankarachariyar Dental College and Vinayaka Mission’s Research Foundation issued the approval (IRC/22102020/S/2).

The minor oral surgical procedures were subcategorized as aerosol and non-aerosol procedures of six small groups comprising five samples in each group like extraction, trans-alveolar extraction, arch bar placement/removal, intra-oral incision, drainage, biopsy, and surgical removal of an impacted tooth. They were also divided into short (less than 30 minutes) and long procedures (more than 30 minutes) depending on duration. There were a total of 30 participants of ages ranging from 20 to 50 years. The details of the study were explained and informed consent was obtained from all the participants.

Preparation of luminol solution

To prepare the luminol solution, 0.1 g of luminol (5-amino-2,3-dihydro-1,4-phthalazinedione) [1] was dissolved in 10 mL of concentrated ammonia and further diluted with 100 mL of water. Before use, 1 mL of the prepared solution was mixed with 4 mL of distilled water and combined with 0.5 mL of 3% H_2_O_2_. Luminol was dissolved in an alkaline mixture with hydrogen peroxide. When exposed to metal ions or metal complexes like hemoglobin, this mixture would generate a vivid blue chemiluminescence. Approximately 2 L of the prepared solution was sufficient to cover an area exceeding 8 m^2^ in the clinical setup.

**Figure 1:**
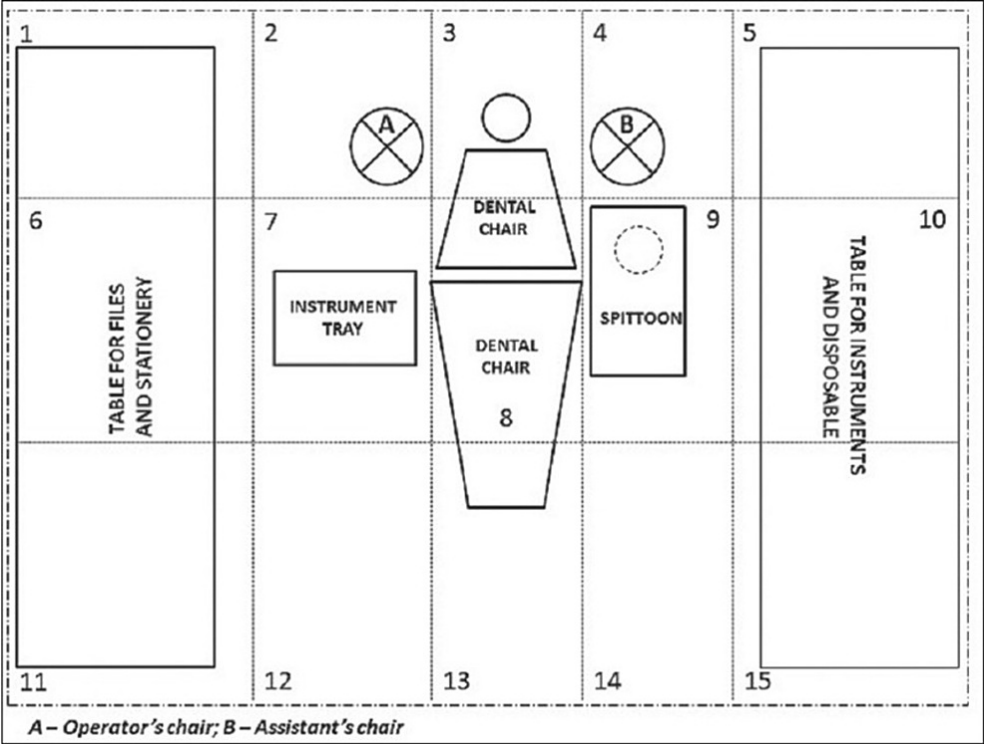
Schematic representation of clinical subsites. Picture courtesy: Al-Eid et al. [2]. 1, 6, and 11: tabletop for files and stationery; 5, 10, and 15: table for instruments and disposable; 2, 3, and 4: flooring behind the dental chair (including the operators' and assistants’ position); 7: instrument spray and handpiece; 8: operating light and dental chair armrest; 9: cuspidor and suction unit; 12, 13, and 14: flooring in front of dental chair.

This procedure was performed in an isolated aseptic closed cubicle. During this procedure, both the operator and assistant wore a PPE kit comprising a head cap, face mask, face shield, and sterile gloves. The patient was covered with disposable aprons. Figure 1 shows the schematic representation of clinical subsites.

Assessment

As part of the standard protocol, pre-procedural rinses using 0.2% chlorhexidine gluconate solution were administered before each oral surgical procedure. Following the completion of the procedure, the patient, surgeon, and assistant were instructed to vacate the area leaving their PPE kits behind.

Subsequently, a prepared luminol solution was sprayed onto the PPE kits and the isolated cubicle area to identify visually imperceptible blood contamination in the room. The shelf life of the luminol solution is 3-5 years. This detection process occurred in complete darkness after turning off all light sources allowing for the observation of emitted chemiluminescence.

**Figure 2:**
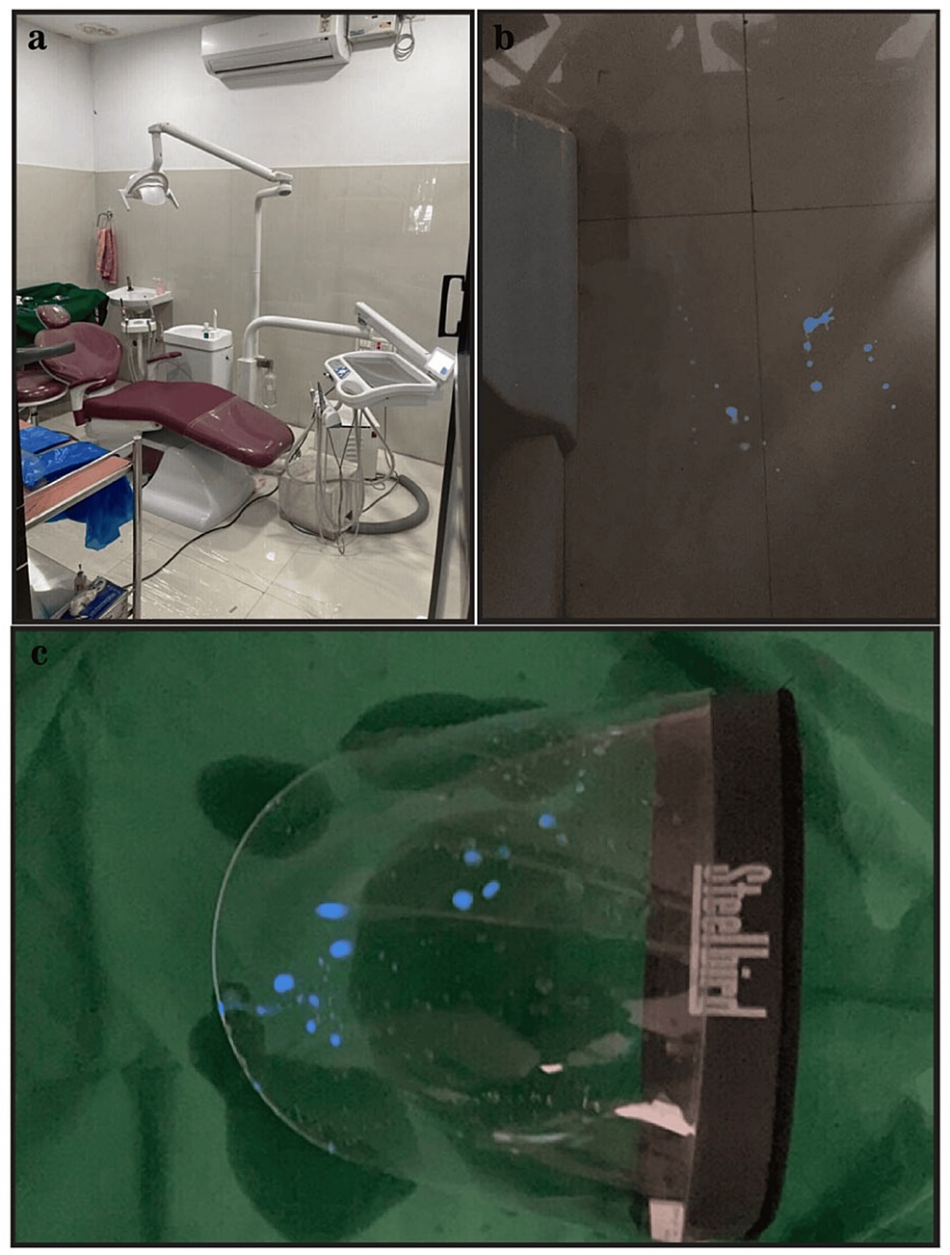
Representation of surgical clinical sites and equipment prone to contamination. (a) Dental clinical subsites: procedures carried out. (b) Floor near the operator: chemiluminescence of luminol denotes visually imperceptible blood contamination. (c) Face shield: chemiluminescence of luminol denotes visually imperceptible blood contamination.

**Figure 3:**
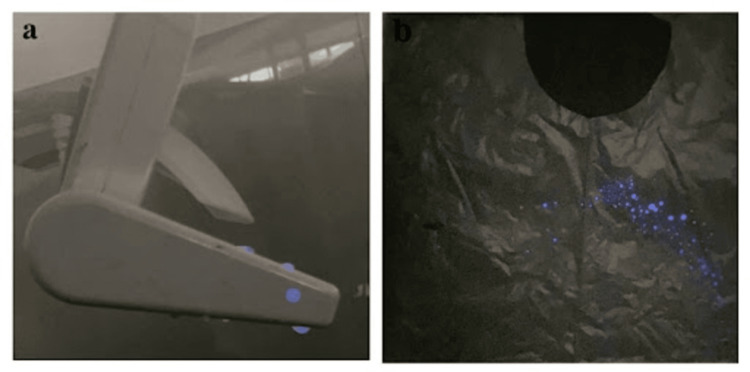
Representation of clinical sites and equipment prone to contamination. (a) Operating light handle: chemiluminescence of luminol denotes visually imperceptible blood contamination. (b) PPE kit: chemiluminescence of luminol denotes visually imperceptible blood contamination.

Any blood spillage in the segmented clinical subsites and on the PPE kits exhibiting chemiluminescence was promptly identified and photographed. It is worth noting that the chemiluminescent effect of luminol lasted for a brief duration of only 20 seconds [2] (Figures 2, 3).

## Results

**Figure 4:**
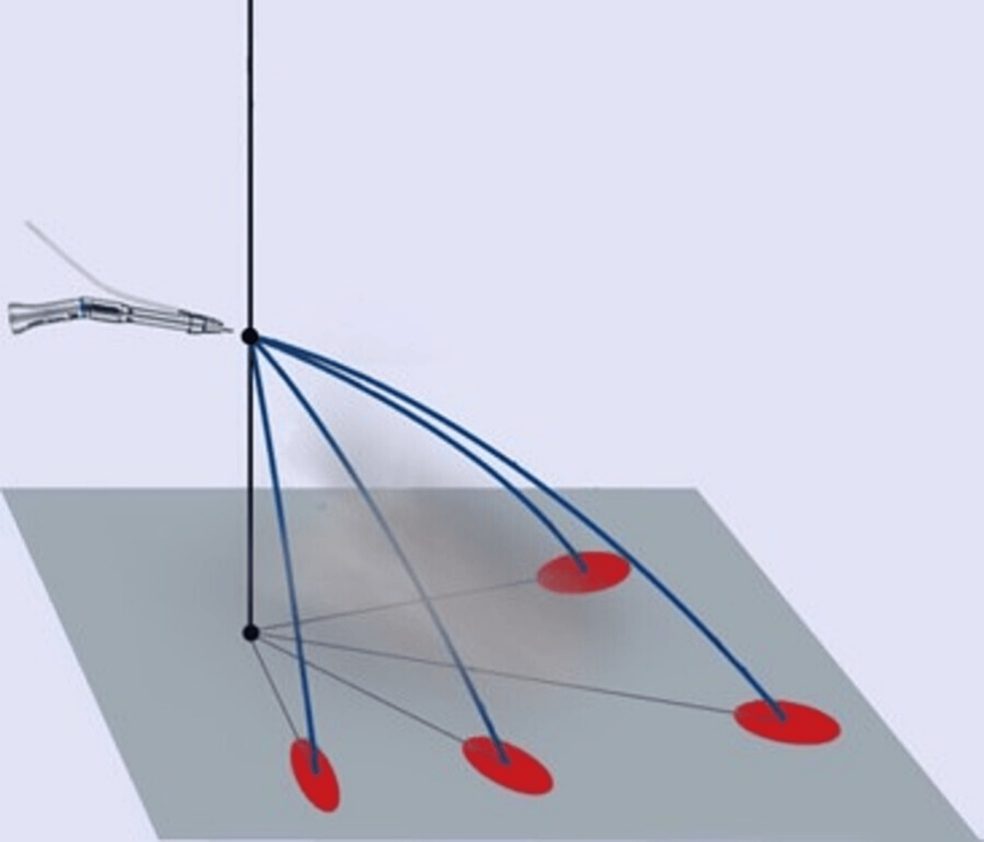
Graphical representation of trajectories of splatter aerosol procedures.

The results of blood splatter among different oral surgical procedures between the non-aerosol and aerosol groups were statistically analyzed using the chi-square test. Blood contamination was examined on the PPE kit and clinical surfaces, and compared with the duration of oral surgical procedures. Figure 4 graphically represents the trajectories of splatter aerosol procedures.

Comparing the duration of oral surgical procedures among non-aerosol (<15 minutes), aerosol (>30 minutes), and non-aerosol (>30 minutes) categories (Tables 1), the detection of imperceptible blood using chemiluminescence on the assistant’s apron (13.3%, 70%, and 100%; p-value = 0.001) (n = 2, 7, 5, 14), assistant’s face shield (6.7%, 60%, and 100%; p-value = 0.001) (n = 1, 6, 5, 12), suction apparatus (20%, 80%, and 80%; p-value = 0.005) (n = 3, 8, 4), floor (26.7%, 80%, and 100%; p-value = 0.003) (n = 4, 8, 5), and walls (0, 50%, and 80%; p-value = 0.001) (n = 0, 5, 4), respectively, showed statistically significant results (Table 2). The p-value has been deemed statistically significant at p < 0.05.

**Table 1:** Comparison between aerosol and non-aerosol procedures based on duration. *p<0.05 is considered statistically significant.

		Type			Total	P value
		Non-aerosol <15 min (n=15)	Aerosol >30 min (n=10)	Non-aerosol >30 min (n=5)		
Patient apron	n	13	10	5	28	0.343
	%	86.7	100.0	100.0	93.3	
Doctor’s apron	n	14	10	5	29	0.596
	%	93.3	100.0	100.0	96.7	
Doctor’s face shield	n	9	9	5	23	0.089
	%	60.0	90.0	100.0	76.7	
Headrest	n	3	4	3	10	0.223
	%	20.0	40.0	60.0	33.3	
Assistant’s apron	n	2	7	5	14	0.001^*^
	%	13.3	70.0	100.0	46.7	
Assistant’s face shield	n	1	6	5	12	0.00^*^
	%	6.7	60.0	100.0	40.0	
Spittoon	n	15	10	5	30	-
	%	100.0	100.0	100.0	100.0	
Suction apparatus	n	3	8	4	15	0.005^*^
	%	20.0	80.0	80.0	50.0	
Instrument tray	n	14	10	5	29	0.596
	%	93.3	100.0	100.0	96.7	
Floor below the suction apparatus	n	4	8	5	17	0.003^*^
	%	26.7	80.0	100.0	56.7	
Armrest of dental chair	n	1	1	0	2	0.765
	%	6.7	10.0	.0	6.7	
Operating light	n	12	9	4	25	0.787
	%	80.0	90.0	80.0	83.3	
Walls near spittoon	n	0	5	4	9	0.001^*^
	%	.0	50.0	80.0	30.0	
Walls near headrest	n	0	3	1	4	0.086
	%	.0	30.0	20.0	13.3	
Floor near surgeon	n	4	5	4	13	0.099
	%	26.7	50.0	80.0	43.3	

**Table 2:** Comparison between aerosol and non-aerosol procedures by types. *p<0.05 is considered statistically significant. Except for the suction apparatus, the contamination difference is non-significant for non-aerosol and aerosol types.

		Types		Total	P value (p<0.05)
		Non-aerosol (n=20)	Aerosol (n=10)		
Patient apron	n	18	10	28	0.540
	%	90.0	100.0	93.3	
Doctor’s apron	n	19	10	29	1.0
	%	95.0	100.0	96.7	
Doctor’s face shield	n	14	9	23	0.372
	%	70.0	90.0	76.7	
Headrest	n	6	4	10	0.690
	%	30.0	40.0	33.3	
Assistant apron	n	7	7	14	0.122
	%	35.0	70.0	46.7	
Assistant face shield	n	6	6	12	0.139
	%	30.0	60.0	40.0	
Spittoon	n	20	10	30	1.0
	%	100.0	100.0	100.0	
Suction apparatus	n	7	8	15	0.05^*^
	%	35.0	80.0	50.0	
Instrument tray	n	19	10	29	1.0
	%	95.0	100.0	96.7	
Floor below the suction apparatus	n	9	8	17	0.119
	%	45.0	80.0	56.7	
Armrest of dental chair	n	1	1	2	1.0
	%	5.0	10.0	6.7	
Operating light	n	16	9	25	0.640
	%	80.0	90.0	83.3	
Walls near spittoon	n	4	5	9	0.115
	%	20.0	50.0	30.0	
Walls near headrest	n	1	3	4	0.095
	%	5.0	30.0	13.3	
Floor near surgeon	n	8	5	13	0.705
		40.0	50.0	43.3	

## Discussion

An infectious disease can be transmitted or spread. Hu et al. [13] proposed that healthcare workers face a high risk of contracting blood-borne diseases like HBV, HIV, and tuberculosis, emphasizing the mandatory need for infection prevention control. Limited research exists on analyzing the risk factors and precautionary measures, specifically related to surgical site infections [14].

Pathogens can be directly transmitted through person-to-person contact like the dispersion of droplets through sneezing, coughing, or spatter generated during dental procedures. Unprotected contact with infectious wounds, abscesses, or contaminated body fluids like blood, saliva, semen, and vaginal secretions is a common route for transmission of infectious agents leading to hepatitis, herpes infection, HIV infection, and tuberculosis.

Dental workers face challenges like exposure to blood and saliva during treatments even when the blood is not visibly apparent in saliva. Indirect transmission occurs when microorganisms are transferred to an object or surface and then conveyed to another person who comes into contact with them. For example, a dental chart handled by a dental assistant wearing contaminated gloves may be later touched by a receptionist with bare hands serving as an example of indirect transmission.

During dental procedures, aerosols, sprays, and splatter may contain blood, saliva, and nasal secretions. Though these terms differ in particle size, they involve droplets of potentially infectious materials [15]. High-speed handpieces (airotor and micromotor) and ultrasonic scalers generate fine aerosols invisible to the naked eye. These particles are less than 10 μm in diameter [15]. Unlike larger-particle spatter which is significant for infection transmission, these fine aerosols can linger in air for extended periods and may be inhaled.

Distinguishing between aerosols and larger-spatter particles is crucial. Inhaling the bacteria present in aerosols is akin to being exposed to someone sneezing in your face twice a minute from a one-foot distance. Moreover, aerosols have the potential to transmit respiratory infections [15].

On the contrary, the use of rotary dental and surgical instruments (e.g., handpieces, ultrasonic scalers) and air-water syringes produces a visible spray. This spray mainly consists of a larger-particle spatter, comprising water, saliva, blood, microorganisms, and various debris [15].

Micik and colleagues [16-21] defined splatter as airborne particles exceeding 50 μm in diameter. They characterized these particles as behaving ballistically, being forcefully expelled from the operating site and following a trajectory similar to a bullet until reaching a surface or descending to the floor. Due to their size, these particles are too large to stay suspended in the air for an extended period and become airborne for a brief period of time. The splatter travels a short distance and rapidly settles, landing on the floor, nearby surfaces in the operatory, dental healthcare personnel providing care, or the patient.

Originally synthesized in 1908, luminol (5-amino-2,3-dihydro-1,4-phthalazine dione) finds primary use at crime scenes. Its application for detecting visually imperceptible blood stains was reported in 1937 following experiments involving hydrogen peroxide and derivatives from the heme group [22]. This forensic luminol operates through a catalytic pathway exhibiting peroxidase-like activity on hemoglobin present in the blood. The reaction results in chemiluminescence emitting light at a wavelength of approximately 428 nm (appearing blue in the visible spectrum). In the context of minor oral surgical procedures, luminol was utilized to identify visually imperceptible blood contamination.

In their 2014 study, Englehardt and his team found that aerosolized and splattered blood could travel approximately 24 inches, thereby potentially contaminating an area as large as a 6-foot radius from the surgical site. This was observed using luminol solution during abdominal surgeries [23]. Additionally, we identified blood splatter over a horizontal area of 3.1 feet and a vertical distance of 4.8 feet from the floor.

Al-Eid et al. [2] concluded that blood contamination due to aerosol procedures was high on surgeons’ apron (73.3%) and assistant apron (66.7%), which were similar to our study irrespective of non-aerosol (surgeon’s apron (95%) and assistant apron (35%)) and aerosol procedures (surgeon’s apron (100%) and assistant apron (70%)).

In 2020, Gandolfi and colleagues emphasized the critical significance of protective measures, particularly PPE in dental practice [24]. They underscored that every patient could potentially carry an infectious disease that might be transmitted through dental aerosols. Consequently, they advocated for the universal adoption of precautions for infection prevention in dental settings.

The COVID-19 pandemic proved the importance of a PPE kit (apron, face shield, N95 mask) for everyone in the healthcare setting to protect from aerosol transmission during treatment. Our study stresses the need for continuing such practices during routine oral surgical procedures.

Limitations

This study's constraints included a small sample size across the groups. Moreover, the luminol solution had a limited transition time of 20 seconds, requiring individual spraying and documentation for each clinical subsite. The chemiluminescence had to be documented within this brief time frame as the chemical illumination was irreversible.

## Conclusions

From our study, we observed that both aerosol procedures within 30 minutes and non-aerosol procedures more than 30 minutes had minute undetectable blood spillage over an area of 3.1 feet horizontally and 4.8 feet vertically from the spittoon. So, we emphasize that a PPE kit is mandatory for both surgeon and assistant while performing oral surgical procedures to prevent such cross infections. Further studies can be done on measuring the size of the splatter and by isolating the organism from the PPE and clinical surfaces for all dental procedures. Also, a questionnaire study can be done among the healthcare workers to find out whether infectious diseases were reported among the healthcare community without any direct contact.
